# Primary care consultations for respiratory tract symptoms during the COVID-19 pandemic: a cohort study including 70,000 people in South West England

**DOI:** 10.1093/fampra/cmab127

**Published:** 2021-10-11

**Authors:** Hajira Dambha-Miller, Hilda O Hounkpatin, Jeffrey Morgan-Harrisskitt, Beth Stuart, Simon D S Fraser, Paul Roderick

**Affiliations:** 1 School of Primary Care, Population Sciences and Medical Education, Faculty of Medicine, University of Southampton, Southampton, United Kingdom; 2 Strategy and Transformation Department, NHS South, Central and West, Eastleigh, United Kingdom

**Keywords:** access to care, consultation, electronic medical records, primary care, public health, respiratory diseases

## Abstract

**Background:**

Primary care consultations for respiratory tract symptoms including identifying and managing COVID-19 during the pandemic have not been characterized.

**Methods:**

A retrospective cohort analysis using routinely collected records from 70,431 adults aged 18+ in South England within the Electronic Care and Health Information Analytics (CHIA) database. Total volume and type of consultations (face-to-face, home visits, telephone, email/video, or out of hours) for respiratory tract symptoms between 1 January and 31 July 2020 (during the first wave of the pandemic) were compared with the equivalent period in 2019 for the same cohort. Descriptive statistics were used to summarize consultations by sociodemographic and clinical characteristics, and by COVID-19 diagnosis and outcomes (death, hospitalization, and pneumonia).

**Results:**

Overall consultations for respiratory tract symptoms increased by 229% during the pandemic compared with the preceding year. This included significant increases in telephone consultations by 250%, a 1,574% increase in video/email consultations, 105% increase in home visits, and 92% increase in face-to-face consultations. Nearly 60% of people who presented with respiratory symptoms were tested for COVID-19 and 16% confirmed or clinically suspected to have the virus. Those with complications including pneumonia, requiring hospitalization, and who died were more likely to be seen in-person.

**Conclusion:**

During the pandemic, primary care substantially increased consultations for respiratory tract symptoms to identify and manage people with COVID-19. These findings should be balanced against national reports of reduced GP workload for non-COVID care.

**Key Messages**

- Consultations for respiratory symptoms increased by 229% during COVID-19 pandemic.
- In-person, phone, home visits, out of hours, and virtual consultations increased.
- 60% of people with respiratory symptoms were tested for SARS-CoV-2.
- Severe complications were prioritized and seen in-person.

## Introduction

Caused by the novel Severe Acute Respiratory Syndrome Coronavirus 2 (SARS-CoV-2), the COVID-19 pandemic has infected 1,617,327 people in the United Kingdom and is responsible for 66,713 deaths as of 30 November 2020. NHS England declared a Level 4 National Incident in January 2020 triggering substantial primary care service reorganization. This prioritized care for people with COVID-19 and aimed to limit viral transmission through digital consultations and physical spaces between and within GP practices. The impact of this rapid service restructuring has varied.^1–4^ There are some suggestions that services were limited with reduced GP workloads.^5^ A number of media reports and a letter from NHS England to all GP practices criticized primary care for limiting services and urgently encouraged resumption of usual care.^6^ Responses from both the British Medical Association and the Royal College of GPs have disputed the suggestion that GP workloads declined during the pandemic, highlighting the impact that this information has had on GP morale and emphasizing that prepandemic primary care workloads were already unsustainable.^7^

Recent observational studies show that overall consultations rates did drop during the pandemic from an average of four per person per year, to less than three once the national lockdown was introduced.^8,9^ However, existing studies have focused exclusively on overall trends and non-COVID-related care such cancer diagnoses, mental health, chronic disease, and immunization programs.^9,10^ Disaggregated data on primary care responsiveness and consultation workload in managing pandemic-related illness have received little attention. There is a lack of studies examining consultation volume and delivery in primary care specific to respiratory tract symptoms, and the identification and management of COVID-19. Primary care services already treat substantial numbers of people with respiratory tract symptoms at a rate of 125–1,110 consultations per 1,000 registered patients, costing the NHS £11,596,350 per year.^11^ It is unclear if further capacity for managing respiratory tract symptoms was generated and if the reported reduction in non-COVID consultations was matched with an increase in pandemic-related clinical workload. This information may help challenge narratives on reduced GP workloads during the pandemic and inform the need for further consultation capacity in primary care as additional COVID-19 waves follow. In this study, we aimed to examine the volume and type of consultations in primary care for respiratory tract symptoms during the first wave of the COVID-19 pandemic.

## Methods

### Study design

A retrospective cohort analysis.

### Data source

The Care and Health Information Analytics (CHIA) is an electronic NHS UK regional database that includes individual-level anonymized live data from primary care records linked to local acute hospital trusts. Data include 1.5 million medical records from consenting patients and have been collected continuously across 160 GP practices covering urban and rural populations in Southern England. This includes READ diagnostic codes for all consultations with a diagnosis or symptoms of respiratory symptoms alongside demographic data, service utilization, investigations, medications, and outcomes from primary care and local hospitals.

### Study population

We identified a cohort of people within the CHIA database aged over 18 years who used primary care services with respiratory symptoms during the first wave of the COVID-19 pandemic (1 January 2020 until 31 July 2020). This included anyone with a Read code for rhinitis; unspecified respiratory tract symptoms; pharyngitis; tonsillitis; acute sinusitis; otitis media; earache; influenza; laryngitis and tracheitis including epiglottitis and croup; or a combination of respiratory symptoms including fever, new cough, productive cough, dry cough, cold symptoms, and sore throat. A full list of Read codes is available from the authors. A continuous, complete, and linked secondary care electronic record over the pandemic period and corresponding prepandemic period in 2019 was required for inclusion.

Within this larger cohort presenting with respiratory symptoms, we identified a second cohort with COVID-19. This included confirmed and suspected cases with complete data. Confirmed cases were defined as a positive RT-PCR assay for SARS-CoV-2 on nasal or pharyngeal swab. Due to limited availability of testing, Public Health England guidance on case definition at the time, advised on the recording of suspected cases based on clinical and radiological findings.^12^ Recent studies have shown that GP clinical suspicion of COVID-19 closely match with confirmed cases for outcomes.^13^ Detail on the electronic record ontology with regard to COVID-19 case definition has been reported in other studies.^14^ Further information and our rationale for case definition can additionally be found in our recent consensus statement.^15^

### Primary care consultations

This was defined as the total number of primary care consultations recorded in the electronic record from the 1 January to 31 July 2020 (during the pandemic) and 1 January to 31 July 2019 (prepandemic). We extracted data on consultation type including face-to-face, home visits, telephone, email/video, or out of hours, where these were available. We focused on total consultation numbers in the cohort rather than per patient consultations.

### Sociodemographic and clinical variables

We used the last recorded entry before the study start date (1 January 2020) to extract age, sex, self-recorded ethnicity, and socioeconomic status with the English Index of Multiple Deprivation (IMD) categorized into quintiles (IMD quintile 1 = most deprived; 5 = least deprived).^16^ For comorbidities, we used conditions coded within the Quality and Outcomes Framework, a financial incentivization scheme in primary care with high levels of accuracy. This included stroke, chronic kidney disease, chronic obstructive pulmonary disease, asthma, Type 1 or 2 diabetes, cancer, mental health disorders, atrial fibrillation, cardiovascular disease, epilepsy, heart failure, and rheumatoid arthritis. For medication use, the last recorded repeat prescription was extracted and included the following drug groups: antiplatelets, anticoagulants, angiotensin-converting enzyme inhibitors, angiotensin receptor blocker, diuretics, calcium-channel blockers, beta blockers, alpha blockers, insulin, oral hypoglycemics, nonopiate analgesics, amitriptyline, statins, nonsteroidal, nonstatin lipid lowering drugs, proton-pump inhibitors, and disease modifying antirheumatic drugs.

### COVID-19 outcomes

Within the COVID-19 cohort, we examined outcomes after a diagnosis of suspected or confirmed SARS-CoV-2 during the follow-up period. For pneumonia, this included Read codes with a clinical diagnosis or radiological evidence of SARS-CoV-2-related pneumonia. Evidence of hospitalization was extracted from the linked secondary care record. We examined all-cause mortality in the records by using linked hospital records and verified through the Office for National Statistics. Deaths were not limited to those in hospital and included any recorded death. We did not look at COVID-19-specific mortality which requires a flag within 28 days of case confirmation, as the exact dates of cases were not sufficiently robust within the dataset, and there was also uncertainty about coding and death certification around COVID-19-specific death in the early part of the pandemic.^17^

### Analysis

The baseline demographic and clinical characteristics were summarized for the cohort at the start of the pandemic as counts (percentages) for categorical variables and means (SDs) for continuous variables. Descriptive statistics were used to summarize consultation volume and type, and we then compared these in the same cohort before the pandemic using chi-squared and *t*-tests as appropriate. Univariate and multivariate mixed-effects logistic regression models (with a random effect on patient ID) were fitted to assess associations between an increase in consultation volume and sociodemographic and clinical variables. Next, we summarized consultation volume and type as percentages among those with COVID-19 who had pneumonia, a hospitalization, or had died. Analyses were undertaken using Stata SE Version 15.0 (StataCorp, College Station, TX, USA).

### Ethical considerations

The study received ethical approval from the University of Southampton and governance approval from the Care and Health Information Exchange Information Governance Group (CHIE IGG). We report our findings in line with STROBE and RECORD guidelines for observational studies using routinely collected health data.

### Data availability

Anonymized individual-level data used in this study were extracted from the CHIA database. Data are available with reasonable request from CHIA.

## Results

### Participant characteristics

Our cohort included 70,431 adults across 160 GP practices in South England who consulted one or more times in primary care for acute respiratory symptoms during the first wave of the pandemic. The mean (SD) age of the cohort was 51 (25.41) years, there were more women than men (59% vs 41%), most were white (54%) and the largest group were from least deprived backgrounds (IMD quintile 5) (29%). A summary of sociodemographic and clinical characteristics is shown in Table 1 alongside missing data. Ethnicity data were missing for 42%, and people with missing data were more likely to be from deprived backgrounds.

**Table 1.** Baseline sociodemographic and clinical characteristics of the CHIA cohort who consulted in primary care for acute respiratory tract symptoms during the COVID-19 pandemic.

Variables	*n* = 70,431
Sex	
Female, *n* (%)	41,279 (58.61)
Male, *n* (%)	29,152 (41.39)
Patients missing data, *n* (%)	0 (0)
Age (years)	
Age, mean (SD)	51.14 (25.41)
Patients missing data, *n* (%)	0 (0)
Ethnicity	
White, *n* (%)	37,974 (53.92)
Asian, *n* (%)	1,746 (2.48)
Black, *n* (%)	365 (0.52)
Mixed or Other, *n* (%)	676 (0.96)
Missing data, *n* (%)	29,670 (42.13)
Index of multiple deprivation	
1, *n* (%) most deprived	8,397 (11.92)
2, *n* (%)	12,301 (17.47)
3, *n* (%)	12,819 (18.2)
4, *n* (%)	15,178 (21.55)
5, *n* (%) least deprived	20,040 (28.45)
Missing data, *n* (%)	1,696 (2.41)
Smoking status	
Current smoker, *n* (%)	9,466 (13.44)
Ex smoker, *n* (%)	21,425 (30.42)
Never smoked, *n* (%)	31,264 (44.39)
Missing data, *n* (%)	8,276 (11.75)
Comorbidities recorded	
Stroke, *n* (%)	3,765 (5.35)
Chronic kidney disease, *n* (%)	5,414 (7.69)
COPD, *n* (%)	5,451 (7.74)
Asthma, *n* (%)	9,422 (13.38)
Diabetes, *n* (%)	7,547 (10.72)
Cancer, *n* (%)	4,972 (7.06)
Mental health disorders, *n* (%)	9,839 (13.97)
Atrial fibrillation, *n* (%)	4,760 (6.76)
Cardiovascular disease, *n* (%)	625 (0.89)
Epilepsy, *n* (%)	960 (1.36)
Heart failure, *n* (%)	2,976 (4.23)
Rheumatoid arthritis, *n* (%)	1,061 (1.51)

COPD, chronic obstructive pulmonary disease.

### Consultations for respiratory tract symptoms by volume and type

There were 103,999 consultations among 70,431 people for respiratory symptoms during the pandemic. This was 229% higher than the equivalent period in the preceding year for respiratory symptoms within the same cohort (*P* < 0.01). Consultation volume was higher across all types with significant increases in telephone (250%) and video/email consultations (1,574%). A higher percentage of consultations were video/email and telephone consultations during the pandemic compared with before the pandemic (9.3% vs 1.8% and 75.7% vs 71.2% for video/email and telephone consultations, respectively). These results are summarized in Table 2. Variations in the sociodemographic and clinical characteristics by total consultation and type are summarized before and during the pandemic in Supplementary Table 1. Compared with the preceding year, there were statistically significant increases in consultations among men (250%) and Asian minority groups (488%). The shift in proportion of the different types of consultations were similar across ethnic and socioeconomic groups, with video consultations making up the largest proportion of consultations for all groups during the pandemic. In-person consultations including face-to-face and home visits significantly (*P* < 0.001) increased among those from higher socioeconomic groups IMD quintile 4 (173%) and quintile 5 (203%). Where convergence was achieved, sociodemographic (except for age) and clinical variables were not significantly associated with an increase in consultation volume in mixed-effects logistic regression models (Supplementary Table 2).

**Table 2.** Summary of consultation volume and type for acute respiratory symptoms before and during the first wave of the COVID-19 pandemic in the same CHIA cohort (*n* = 70,431).

Activity type	Before the pandemic: January–July 2019	During the pandemic: January–July 2020	% increase
Total number of consultations/encounters	*n* = 31,574	*n* = 103,999	229
Face-to-face at the surgery	3,678 (11.6%)^a^	7,066 (6.8%)	92
Home visit	2,474 (7.8%)	5,078 (4.9%)	105
Out of hours contact	2,358 (7.5%)	3,470 (3.3%)	47
Telephone consultation	22,484 (71.2%)	78,677 (75.7%)	250
Video/email consultation	580 (1.8%)	9,708 (9.3%)	1,574

^a^Column percentages presented in brackets.

### Primary care consultations among people with COVID-19

Within this cohort of people with respiratory symptoms, 41,516 (58.95%) were tested for COVID-19; 774 (1.86%) were confirmed on RT-PCR and 6,147 (14.8%) were coded as clinically suspected cases. Complete consultation data were only available for 401 confirmed cases on RT-PCR and 4,489 suspected cases. We combined these to generate a COVID-19 cohort of 4,890 people and carried out complete case analysis. This cohort consulted primary care a total of 14,489 times during the pandemic and were primarily managed through telephone triage (75%). The mean age was 55.7 (25) years, and they were mostly white, female and from the least deprived IMD quintiles with few or no multimorbidities. This is summarized in Table 3.

**Table 3.** Summary of consultations among the CHIA COVID-19 cohort by sociodemographic and clinical characteristics (*n* = 4,890).

Variables	Total consultations	Face-to-face at the practice	Home visit	Out of hours	Telephone encounter	Video/email
Number of consultations	14,489	672	601	388	10,891	1,937
Sex						
Female, *n* (%)	8,706 (60.1)	424 (63.1)	332 (55.2)	212 (54.6)	6,501 (59.7)	1,237 (63.9)
Male, *n* (%)	5,783 (39.9)	248 (36.9)	269 (44.8)	176 (45.4)	4,390 (40.3)	700 (36.1)
Missing, *n* (%)	0	0	0	0	0	0
Age						
Age, mean (SD)	55.7 (25)	53.7 (23.5)	78.8 (13.5)	56.5 (28)	53.8 (24.4)	55.7 (26.3)
Missing, *n* (%)	0	0	0	0	0	0
Ethnicity						
Asian, *n* (%)	505 (3.5)	12 (1.8)	2 (0.3)	1 (0.3)	424 (3.9)	66 (3.4)
Black, *n* (%)	95 (0.7)	3 (0.4)	9 (1.5)	3 (0.8)	62 (0.6)	18 (0.9)
Mixed or Other, *n* (%)	100 (0.7)	6 (0.9)	1 (0.2)	1 (0.3)	80 (0.7)	12 (0.6)
White, *n* (%)	6,460 (44.5)	352 (52.4)	279 (46.4)	179 (46.1)	4,476 (41.1)	1,174 (60.7)
Missing, *n* (%)	7,329 (50.6)	299 (44.5)	310 (51.6)	204 (52.5)	5,849 (53.7)	667 (34.4)
Index of multiple deprivation						
1, *n* (%) most deprived	1,400 (9.7)	61 (9.1)	55 (9.2)	66 (17)	1,053 (9.7)	165 (8.5)
2, *n* (%)	2,797 (19.3)	102 (15.2)	129 (21.4)	93 (24)	2,120 (19.5)	353 (18.2)
3, *n* (%)	2,804 (19.3)	108 (16.1)	97 (16.1)	69 (17.7)	2,113 (19.4)	417 (21.6)
4, *n* (%)	3,273 (22.6)	168 (25)	132 (22)	72 (18.6)	2,533 (23.3)	368 (19)
5, *n* (%) least deprived	3,974 (27.4)	219 (32.5)	187 (31.1)	83 (21.4)	2,880 (26.3)	605 (31.2)
Missing, *n* (%)	241 (1.7)	14 (2.1)	1 (0.2)	5 (1.3)	192 (1.8)	29 (1.5)
Smoking status						
Current smoker, *n* (%)	1,536 (10.6)	61 (9.1)	35 (5.8)	53 (13.7)	1,212 (11.1)	175 (9)
Ex smoker, *n* (%)	5,379 (37.1)	215 (32)	247 (41.1)	140 (36.1)	4,099 (37.5)	678 (35)
Never smoked, *n* (%)	6,575 (45.4)	357 (53.1)	308 (51.3)	158 (40.7)	4,850 (44.5)	902 (46.6)
Missing, *n* (%)	999 (6.9)	39 (5.8)	11 (1.8)	37 (9.5)	730 (6.7)	182 (9.4)
Comorbidity^a^						
0	5,649 (39)	275 (40.9)	145 (24.1)	144 (37.1)	4,245 (39)	840 (43.4)
1	4,058 (28)	203 (30.2)	149 (24.8)	107 (27.6)	3,098 (28.4)	501 (25.8)
2	1,787 (12.4)	86 (12.8)	79 (13.1)	45 (11.6)	1,280 (11.8)	297 (15.3)
3	1,228 (8.5)	49 (7.3)	72 (12)	34 (8.8)	907 (8.3)	166 (8.6)
4	847 (5.8)	21 (3.1)	81 (13.5)	28 (7.2)	632 (5.8)	85 (4.4)
≥5	920 (6.3)	38 (5.7)	75 (12.5)	30 (7.7)	729 (6.7)	48 (2.5)
Missing, *n* (%)	0	0	0	0	0	0

This table reflects consultations and some patients might be represented more than once. COPD, chronic obstructive pulmonary disease.

^a^Comorbidities included stroke, chronic kidney disease, COPD, asthma, Type 1 or 2 diabetes, cancer, mental health disorders, atrial fibrillation, cardiovascular disease, epilepsy, heart failure, and rheumatoid arthritis.

### Primary care consultations and COVID-19 outcomes

We examined outcomes within the follow-up period as recorded in the electronic record after a code of clinically suspected or RT-PCR confirmed COVID-19; 1,100 (22%) people had pneumonia recorded by the GP, 307 (6%) were hospitalized, and 925 (18%) died from any cause. In terms of consultations, where data were available, we found that this cohort with complications were more likely to be seen in-person through home visits or face-to-face consultations at the practice, compared with the whole cohort. Both telephone and email/video consults were also substantially lower in this group when compared with the whole cohort (*P* < 0.001). A high number of people also consulted with primary care through the out of hours primary care services (18.8%). Table 4 summarizes primary care consultations by COVID-19 outcomes.

**Table 4.** Primary care consultations in the CHIA COVID-19 cohort by outcomes (*n* = 4,890 people with confirmed or suspected COVID-19).

Variables	Total consultations	Face-to-face at the practice	Home visits	Out of hours contact	Telephone encounter	Video/email consultation
Consultations number	14,489	672	601	388	10,891	1,937
All-cause mortality, *n* (%)	1,172 (8.1)	84 (12.5)	182 (30.3)	69 (17.8)	635 (5.8)	202 (10.4)
Hospitalization, *n* (%)	390 (2.7)	44 (6.5)	45 (7.5)	23 (5.9)	243 (2.2)	35 (1.8)
Pneumonia, *n* (%)	1,423 (9.8)	144 (21.4)	96 (16)	51 (13.1)	947 (8.7)	185 (9.6)
Other/not known, *n* (%)^a^	11,504 (79.4)	400 (59.6)	278 (46.2)	245 (63.2)	9,066 (83.3)	1,515 (78.2)

This table represents consultations numbers and some patients might be represented more than once. Each outcome is not mutually exclusive.

^a^We found that 11,504 records had an outcome recorded for COVID-19 but no detail provided to allow us to ascertain what this outcome was. We have included this number as other/not known in the table for completion.

## Discussion

### Key findings

Our data suggest that during the COVID-19 pandemic, primary care managed a high volume of consultations for people with respiratory tract symptoms compared with the equivalent period last year in this same cohort. Related consultation workload increased for in-person, telephone, home visits, out of hours, and video/email consultations. Nearly 60% of those with respiratory symptoms were tested for SARS-CoV-2, and severe complications (pneumonia, hospitalization, or death) were prioritized for in-person consultations.

### Comparison to existing literature

National data show that overall primary care consultations dropped during the pandemic. De Lusignan et al. report that the rate of consultations declined by 27.1% (from 59,431 to 43,324) while a Health Foundation report suggests a 30% decrease per week during the lockdown period.^8,9^ Other studies similarly show reductions in routine primary care including a decline in mental health, chronic disease, and cancer-related workload.^9,10^ Together with media reports, this has led to suggestions that GP practices were closed, and workloads reduced during the pandemic. There have been concerns about the impact of this on routine care. However, previous studies have not quantified workload related specifically to respiratory tract symptoms during pandemic. Across primary and secondary care, services were encouraged to rapidly reorganize and prioritize the management of COVID-19. Our results show that primary care responded with an increase in capacity and managed high through-flow of consultations for people with respiratory symptoms including testing and identification of those with greatest clinical need in terms of COVID-19 outcomes.

Telephone consultations increased by 250% (from 22,484 to 78,677) compared with the previous year which is consistent with national year by year trends toward more telephone triage.^7^ The notable rise in email/video consultations (1,574% from 580 to 9,708) has not previously been seen largely due to concerns about efficiency and they often require subsequent telephone or in-person consultations.^18^ The observed increase in digital consultation usage during the pandemic suggests that views might be changing toward greater acceptability of their use in practice. The impact of digital consultation on clinical care and outcomes, however, remains unclear.^19^ Our data show that digital consultations were more common in people who were younger and healthy (i.e. fewer comorbidities), and unlikely to be employed by GPs where severe COVID-19 outcomes were considered. In-person consultations and home visits were prioritized for those who were most unwell from COVID-19 including people with pneumonia, hospitalization, or who subsequently died. Our findings further corroborate recent evidence showing that primary care can appropriately and efficiently identify patients with complex and serious clinical need, despite growing workloads and the unprecedented impact of COVID-19.^9^ Overall during the pandemic, we also observed a greater number of consultations among people over 50 years compared with younger patients, and those from higher IMD quintiles (3 and 4).^9^ These findings are not consistent with observational studies prior to the pandemic which show higher consultation rates tend to occur in lower socioeconomic groups related to increased comorbidities, health inequalities, and social isolation.^7,20,21^

### Strengths and limitations

Our study sample is drawn from a large database that included 160 GP practices in South England; it reflects both urban and rural areas with heterogeneity in age, sex, and disease profiles. The cohort does include low representation of ethnic minority participants and high number of patients from IMD 4/5 (high sociodemographic backgrounds) which reflects the make-up of this area in England, which may not be generalizable to other parts of the country with more diverse populations. As with other studies using electronic health records, there was also a significant amount of missing data on ethnicity. Moreover, our sample was restricted to people with linked hospital records and complete consultation notes over the study period. Those with incomplete records were not reflected in our sample. It is possible that the characteristics and outcomes of this group may be different to those in the included sample. Our study looked at electronic records of consultations, but it is possible that additional telephone calls, consultations, or clinical workload such may have occurred but were not captured on the electronic records. The workload capture here may be an underestimate. The data we used are drawn from a single large database of routinely clinical records which are not designed to be at “research standard” and will have variations in entries and coding that is dependent on individual clinicians. To some extent, this bias is reduced by the size of the database and we tried to include only standardized coding that is used for payment and administrative purposes which are likely to be of high quality. We did not account for changes in sociodemographic (e.g., IMD quintile) and clinical variables (e.g. smoking, medication) in our regression models as we did not extract prepandemic data on these variables. Finally, the case definition of COVID-19 has been changing over the course of the pandemic especially as testing becomes more available. We included laboratory confirmed cases alongside clinically suspected cases as our COVID-19 cohort in line with Public Health England guidance at the time. It is possible that clinical symptoms may not have been consistently recorded and given that COVID-19 codes are relatively new to practice, uptake may not have been consistent. Further, people who were asymptomatic, with mild symptoms or those admitted directly to hospital who did not present to primary care will be under-represented in our cohort. We also did not include people who presented with nonrespiratory symptoms during the pandemic. As the pandemic progresses, additional symptoms related to COVID-19 are being established including for example, gastrointestinal and dermatological symptoms.^22^ These have not been considered in our study.

### Implications for research and practice

Although non-COVID-related care reduced during the pandemic, our findings show that primary care was open and delivering unprecedented volumes of consultations that prioritized people with respiratory tract symptoms, and those with severe complications of COVID-19. The rapid reorganization of consultation delivery to increased telephone, video, and email consultations may have contributed toward necessary additional capacity that should be acknowledged alongside the decline in routine non-COVID care. Some of these new approaches to consultations that were observed could be taken forward to manage routine workloads for other conditions and allow better integration across service providers and improved delivery of care. However, further work is needed to better understand this increase in consultations. Firstly, further studies using different populations are needed to explore whether this shift in consultations results in greater health inequalities in other areas, particularly those with higher proportion of ethnic minorities or socially disadvantaged populations and characteristics associated with change in consultation volume. It will also be helpful to examine how findings vary across different types of practices. Future studies with data on the number of consultations per event/presentation can also explore whether triaging and remote consulting lead to increased number of consultations for the same event or a shift in care delivery between secondary and primary care.^23,24^ Further research to examine the effectiveness of these consultations in terms of clinical outcomes, as well as cost-effectiveness, and its impact (either positive or negative) on continuity of care is still required. Finally, longer-term analysis is needed to assess the impact on outcomes arising from potentially missed non-COVID diagnoses through reduced face-to-face consultation for other problems.

## Funding

HDM has received National Institute of Health Research School of Primary Care Research (NIHR SPCR) funding (SPCR2014-10043) and Medical Research Council (MRC) funding (MR/V027778/1) to support her COVID-19 research. The views and opinions expressed by authors in this publication are those of the authors and do not necessarily reflect those of the UK National Institute for Health Research (NIHR) or the Department of Health and Social Care.

## Ethical approval

The study received ethical approval from the University of Southampton and governance approval from the Care and Health Information Exchange Information Governance Group (CHIE IGG).

## Conflict of interest

None declared.

## Supplementary Material

cmab127_suppl_Supplementary_Material
