# Supplementary material for: Primary care consultations for respiratory tract symptoms during the COVID-19 pandemic: a cohort study including 70,000 people in South West England
Source: Fam Pract. 2021 Oct 11;39(3):440–6. doi: 10.1093/fampra/cmab127 (PMC9155167; doi:10.1093/fampra/cmab127)
Supplement: cmab127_suppl_Supplementary_Material [file cmab127_suppl_supplementary_material.pdf]

Supplementary Table 1: Summary of consultations for acute respiratory symptoms by sociodemographic and clinical characteristics before and during the COVID-19 pandemic in the same cohort (n=70431)

	**TOTAL CONSULTATIONS**		**FACE-TO-FACE AT THE PRACTICE**		**HOME VISITS**		**OUT OF HOURS**		**VIDEO/EMAIL**		**TELEPHONE**	
	**Before pandemic**	**During pandemic**	**Before pandemic**	**During pandemic**	**Before pandemic**	**During pandemic**	**Before pandemic**	**During pandemic**	**Before pandemic**	**During pandemic**	**Before pandemic**	**During pandemic**
Number of consultations	31574	103999	3678	7066	2474	5078	2358	3470	580	9708	22484	78677
Sex												
Female n (%)	19825 (62.8)	62725 (60.3)	2285 (62.1)	4300 (60.9)	1597 (64.6)	2939 (57.9)	1494 (63.4)	1932 (55.7)	419 (72.2)	6622 (68.2)	14030 (62.4)	46932 (59.7)
Male n (%)	11749 (37.2)	41274 (39.7)	1393 (37.9)	2766 (39.1)	877 (35.4)	2139 (42.1)	864 (36.6)	1538 (44.3)	161 (27.8)	3086 (31.8)	8454 (37.6)	31745 (40.4)
Missing n (%)	0 (0)	0 (0)	0 (0)	0 (0)	0 (0)	0 (0)	0 (0)	0 (0)	0 (0)	0 (0)	0 (0)	0 (0)
Age												
Age mean (SD)	58.7 (25.3)	56.9 (25.2)	55.4 (24.5)	55.2 (24.2)	78.6 (15.7)	78.3 (14.7)	52.1 (28.2)	55.4 (27.3)	42.8 (15.5)	51.6 (24.9)	58 (24.9)	55.7 (25.0)
Missing n (%)	0 (0)	0 (0)	0 (0)	0 (0)	0 (0)	0 (0)	0 (0)	0 (0)	0 (0)	0 (0)	0 (0)	0 (0)
Ethnicity												
Asian n (%)	231 (0.7)	1359 (1.3)	39 (1.1)	47 (0.7)	11 (0.4)	19 (0.4)	16 (0.7)	13 (0.4)	10 (1.7)	231 (2.4)	155 (0.7)	1049 (1.4)
Black n (%)	79 (0.3)	257 (0.3)	5 (0.1)	16 (0.2)	11 (0.4)	15 (0.3)	5 (0.2)	6 (0.2)	6 (1.0)	50 (0.5)	52 (0.2)	170 (0.2)
Mixed or Other n (%)	121 (0.4)	532 (0.5)	4 (0.1)	32 (0.5)	8 (0.3)	39 (0.8)	11 (0.5)	10 (0.3)	1 (0.2)	107 (1.1)	97 (0.4)	344 (0.4)
White n (%)	9753 (30.9)	33169 (31.9)	684 (18.6)	1538 (21.8)	1319 (53.4)	2787 (54.8)	1023 (43.4)	1335 (38.5)	444 (76.6)	5471 (56.4)	6283 (27.9)	22038 (28.0)
Missing n (%)	21390 (67.7)	68682 (66.0)	2946 (80.1)	5433 (76.8)	1125 (45.5)	2218 (43.7)	1303 (55.2)	2106 (60.6)	119 (20.5)	3849 (39.6)	15897 (70.8)	55076 (70.0)

IMD												
1 n (%) most deprived	5435 (17.2)	17976 (17.3)	1052 (28.7)	1900 (26.9)	288 (11.6)	648 (12.8)	531 (22.5)	755 (21.8)	43 (7.4)	1271 (13.1)	3521 (15.7)	13402 (17.0)
2 n (%)	7710 (24.4)	24560 (23.6)	980 (26.6)	1514 (21.4)	565 (22.8)	951 (18.7)	481 (20.4)	778 (22.3)	81 (14.0)	2038 (21.0)	5603 (24.9)	19279 (24.5)
3 n (%)	6023 (19.1)	20374 (19.6)	729 (19.8)	1075 (15.2)	457 (18.5)	941 (18.5)	406 (17.2)	630 (18.2)	45 (7.8)	1915 (19.7)	4386 (19.5)	15813 (20.1)
4 n (%)	5822 (18.4)	19801 (19.0)	449 (12.2)	1230 (17.4)	464 (18.8)	1098 (21.6)	464 (19.7)	675 (19.5)	101 (17.4)	1850 (19.1)	4344 (19.3)	14948 (19.0)
5 n (%) least deprived	6030 (19.1)	19650 (18.9)	401 (10.9)	1219 (17.3)	678 (27.4)	1396 (27.5)	406 (17.2)	581 (16.7)	287 (49.4)	2398 (24.7)	4258 (18.9)	14056 (17.9)
Missing n(%)	554 (1.8)	1638 (1.6)	67 (1.8)	128 (1.8)	22 (0.9)	44 (0.9)	70 (3.0)	51 (1.5)	23 (4.0)	236 (2.4)	372 (1.7)	1179 (1.5)
Smoking												
Current Smoker n (%)	4640 (14.7)	15730 (15.1)	678 (18.4)	1351 (19.1)	180 (7.3)	424 (8.3)	469 (19.9)	581 (16.7)	66 (11.4)	1296 (13.3)	3247 (14.4)	12078 (15.4)
Ex-Smoker n (%)	11351 (36.0)	37729 (36.3)	1357 (36.9)	2436 (34.5)	1041 (42.1)	2224 (43.8)	726 (30.8)	1156 (33.4)	178 (30.6)	3024 (31.2)	8049 (35.8)	28889 (36.7)
Never Smoked n (%)	13668 (43.2)	44338 (42.6)	1447 (39.3)	2904 (41.1)	1210 (48.9)	2364 (46.6)	912 (38.7)	1414 (40.7)	331 (57.1)	4771 (49.1)	9768 (43.5)	32885 (41.8)
Missing n (%)	1915 (6.1)	6202 (6.0)	196 (5.4)	375 (5.3)	43 (1.7)	66 (1.3)	251 (10.6)	319 (9.2)	5 (0.9)	617 (6.4)	1420 (6.3)	4825 (6.1)
Total co-morbidities												
0	8279 (26.2)	28381 (27.3)	1011 (27.4)	2011 (28.5)	405 (16.4)	685 (13.5)	754 (32.0)	1161 (33.4)	262 (45.1)	3738 (38.5)	5847 (26.0)	20786 (26.5)
1	7382 (23.4)	25069 (24.1)	940 (25.6)	1803 (25.5)	446 (18.0)	938 (18.5)	520 (22.1)	839 (24.2)	186 (32.1)	2647 (27.2)	5290 (23.5)	18842 (23.9)
2	4890 (15.5)	17232 (16.6)	566 (15.4)	1236 (17.5)	299 (12.1)	774 (15.2)	404 (17.1)	488 (14.1)	96 (16.6)	1452 (15.0)	3525 (15.7)	13282 (16.9)

3	3937 (12.5)	12892 (12.4)	407 (11.1)	746 (10.6)	377 (15.2)	814 (16.0)	259 (11.0)	398 (11.5)	26 (4.5)	873 (9.0)	2868 (12.8)	10061 (12.8)
4	3169 (10.0)	9058 (8.7)	347 (9.4)	547 (7.7)	360 (14.6)	734 (14.5)	182 (7.7)	279 (8.0)	6 (1.0)	475 (4.9)	2274 (10.1)	7023 (8.9)
>=5	3917 (12.4)	11367 (10.9)	407 (11.1)	723 (10.2)	587 (23.7)	1133 (22.3)	239 (10.1)	305 (8.8)	4 (0.7)	523 (5.4)	2680 (11.9)	8683 (11.0)
Missing n (%)	0 (0)	0 (0)	0 (0)	0 (0)	0 (0)	0 (0)	0 (0)	0 (0)	0 (0)	0 (0)	0 (0)	0 (0)
Total medications												
0	5486 (17.4)	20815 (20)	742 (20.2)	1575 (22.4)	104 (4.2)	212 (4.2)	603 (25.6)	851 (24.4)	207 (35.7)	3092 (31.9)	3830 (17.0)	15085 (19.2)
1	1553 (4.9)	5934 (5.7)	225 (6.1)	505 (7.1)	41 (1.7)	99 (1.9)	125 (5.3)	183 (5.3)	49 (8.4)	845 (8.7)	1113 (5.0)	4302 (5.5)
2	1022 (3.2)	3712 (3.6)	139 (3.8)	279 (3.9)	38 (1.5)	58 (1.1)	72 (3.1)	106 (3.1)	47 (8.1)	439 (4.5)	726 (3.2)	2830 (3.6)
3	641 (2.0)	2495 (2.4)	89 (2.4)	161 (2.3)	26 (1.1)	80 (1.6)	55 (2.3)	89 (2.6)	24 (4.1)	293 (3.0)	447 (2.0)	1872 (2.4)
4	620 (2.0)	2205 (2.1)	68 (1.8)	155 (2.2)	35 (1.4)	71 (1.4)	51 (2.2)	96 (2.8)	14 (2.4)	206 (2.1)	452 (2.0)	1677 (2.1)
>=5	22252 (70.5)	68838 (66.2)	2415 (65.7)	4391 (62.1)	2230 (90.1)	4558 (89.8)	1452 (61.5)	2145 (61.8)	239 (41.1)	4833 (49.8)	15916 (70.8)	52911 (67.2)
Missing n (%)	0 (0)	0 (0)	0 (0)	0 (0)	0 (0)	0 (0)	0 (0)	0 (0)	0 (0)	0 (0)	0 (0)	0 (0)

*Co-morbidities included Stroke, Chronic Kidney Disease, COPD, Asthma, Type 1 or 2 Diabetes, Cancer, Mental Health Disorders, Atrial Fibrillation, Cardiovascular Disease, Epilepsy, Heart Failure and Rheumatoid Arthritis

** Medication included the last recorded repeat prescriptions and included anti-platelets, anti-coagulants, Angiotensin Converting Enzyme-Inhibitors, Angiotensin Receptor Blocker, Diuretics, Calcium-channel blockers, Beta blockers, Alpha blockers, Insulin, Oral hypoglycaemics, Non-opiate analgesics, Amitriptyline, Statins, NSAID, non-statin lipid lowering drugs, Proton-pump inhibitors and DMARDS.

This table reflects consultation numbers; some patients might be represented more than once

Supplementary Table 2. Univariate and multivariate mixed model regression results

	**All consultations (n=9189)**		**Face to Face (n=796)**		**Home visits (n=566)**		**Out of hours (n=115)**		**Video/email (n=378)**		**Telephone (n=6686)**	
	**Unadjusted OR (95% CI)**	**Adjusted OR (95% CI)**	**Unadjusted OR (95% CI)**	**Adjusted OR (95% CI)**	**Unadjusted OR (95% CI)**	**Adjusted OR (95% CI)**	**Unadjusted OR (95% CI)**	**Adjusted OR (95% CI)**	**Unadjusted OR (95% CI)**	**Adjusted OR (95% CI)**	**Unadjusted OR (95% CI)**	**Adjusted OR (95% CI)**
Gender	0.96 (0.78-1.19)	0.92 (0.82-1.05)	0.87 (0.15-5.19)	0.84 (0.61-1.15)	1.19 (0.41-3.45)	1.21 (0.81-1.80)	0.96 (0.00-256.74)	0.57 (0.20-1.69)	1.04 (0.61-1.75)	0.94 (0.59-1.51)	0.99 (0.52-1.92)	0.94 (0.84-1.04)
Age	1.01 (1.00-1.01)	1.00 (1.00-1.01)	1.01 (0.99-1.02)	1.00 (0.99-1.01)	1.00 (0.99-1.01)	0.99 (0.98-1.01)	0.98 (0.96-1.01)	0.98 (0.95-1.02)	1.00 (1.00-1.01)	1.00 (0.99-1.02)	1.01 (1.01-1.01)	1.00 (1.00-1.01)
Ethnicity												
White	1	1	1	1	1	1	1	1	1	1	1	1
Asian	0.93 (0.55-1.59)	1.09 (0.61-1.94)	6.43 (0.36-114.97)	11.12 (0.60-206.24)	5.92 (0.53-66.2)	6.96 (0.59-81.6)	0.68 (0.00-1.98e+08)	0.48 (0.03-8.69)	1 (empty)	1.62e-06 (0-empty)	1.11 (0.58-2.12)	1.26 (0.73-2.17)
Black	1.22 (0.43-3.45)	1.35 (0.51-3.59)	1.00 (empty)	1(empty)	1 (empty)	1 (empty)	1.97e+08 (0 - empty)	empty	0.86 (0.55-1.34)	0.94 (0.58-1.50)	0.72 (0.17-3.04)	0.80 (0.29-2.15)
Mixed or Other	0.88 (0.41-1.89)	1.12 (0.52-2.41)	empty	empty	1.48 (0.13-16.56)	2.04 (0.17-24.6)	empty	empty	1 (empty)	1.84e-06 (0-empty)	1.05 (0.44-2.52)	1.41 (0.61-3.27)
Index of Multiple Deprivation												
1 (Most deprived)	1	1	1	1	1	1	1	1	1	1	1	1
2	0.93 (0.67-1.27)	0.89 (0.74-1.07)	0.63 (0.01-27.93)	0.70 (0.48-1.03)	0.86 (0.46-1.61)	0.84 (0.43-1.61)	1.42 (1.74e-07 - 1.15e+07)	1.76 (0.39-7.90)	0.92 (0.32-2.63)	0.93 (0.49-1.77)	0.96 (0.69-1.32)	0.93 (0.78-1.09)
3	0.75 (0.26-2.17)	0.75 (0.57-0.98)	0.53 (0.00-102.00)	0.62 (0.39-0.98)	1.07 (0.56-2.04)	1.13 (0.57-2.24)	1.68 (1.20e-10-2.34e+10)	4.74 (0.88-25.62)	0.69 (0.02-25.54)	0.72 (0.34-1.50)	0.72 (0.08 - 6.64)	0.75 (0.63-0.89)
4	0.63 (0.12-3.47)	0.65 (0.46-0.92)	0.63 (0.01-29.83)	0.78 (0.47-1.31)	1.01 (0.52-1.96)	1.07 (0.52-2.18)	0.54 (1.93e-13 - 1.50e+12)	0.83 (0.21-3.30)	1.22 (0.14-10.39)	1.09 (0.53-2.26)	0.62 (0.03-14.96)	0.68 (0.57-0.81)
5 (Least deprived)	0.46 (0.02-8.77)	0.52 (0.32-0.84)	0.66 (0.02-20.82)	0.76 (0.40-1.44)	0.74 (0.41-1.37)	0.83 (0.43 - 1.63)	1.01 (0.24-4.28)	1.49 (0.40-5.54)	0.97 (0.42-2.29)	0.92 (0.41-2.06)	0.43 (0.00-123.52)	0.61 (0.51-.73)
Smoking status												
Current smoker	1	1	1	1	1	1	1	1	1	1	1	1
Ex-smoker	0.93 (0.67-1.31)	0.96 (0.81-1.14)	0.84 (0.07-10.62)	0.94 (0.62-1.42)	1.20 (0.31-4.62)	1.43 (0.64-3.18)	0.14 (2.62e-11 - 7.42e+08)	0.17 (0.03-1.04)	1.08 (0.50-2.31)	0.94 (0.49-1.80)	1.03 (0.85-1.25)	1.00 (0.85-1.17)
Never smoked	0.91 (0.58-1.42)	0.97 (0.82-1.15)	0.70 (0.00-130.53)	0.80 (0.53-1.21)	1.01 (0.46-2.20)	1.32 (0.59 - 2.99)	0.19 (1.91e-09 - 1.95e+10)	0.18 (0.03-1.01)	0.62 (0.05-8.40)	0.55 (0.28-1.07)	0.98 (0.04-5.01)	0.68 (0.52-0.88)
Total comorbidities												

0	1	1	1	1	1	1	1	1	1	1	1	1
1	1.38 (0.20-9.44)	1.25 (1.01-1.56)	1.80 (0.00-3541.67)	1.46 (0.96-2.21)	1.45 (0.16-13.20)	1.37 (0.66-2.83)	1.26 (0.55-2.87)	1.41 (0.56-3.57)	1.62 (0.02 - 105.35)	1.51 (0.75-3.05)	1.27 (0.68-2.38)	1.09 (0.94-1.27)
2	1.53 (0.12-19.2)	1.44 (1.06-1.96)	1.97 (0.00-13282.58)	1.73 (1.05-2.85)	1.56 (0.11-21.39)	1.43 (0.65 -3.13)	0.71 (0.22-2.27)	0.75 (0.17-3.24)	1.72 (0.02-191.71)	1.59 (0.71-3.56)	1.50 (0.53-4.27)	1.30 (1.10-1.54)
3	1.56 (0.11-22.0)	1.42 (1.04-1.94)	2.28 (0.00-103139.80)	1.87 (1.04-3.36)	1.41 (0.18-11.01)	1.45 (0.69-3.05)	0.20 (0.02-1.95)	0.31 (0.02-4.46)	1.38 (0.08-22.69)	1.07 (0.46-2.52)	1.72 (0.43-6.88)	1.37 (1.13-1.65)
4	1.41 (0.18-10.9)	1.23 (0.95-1.60)	1.17 (0.15-9.11)	0.96 (0.51-1.83)	1.30 (0.25-6.92)	1.32 (0.60 - 2.89)	empty	empty	1.87 (0.01-463.71)	1.58 (0.61-4.09)	1.79 (0.41-7.89)	1.37 (1.11-1.69)
5	1.56 (0.11-21.4)	1.36 (1.01-1.83)	1.47 (0.01-197.73)	1.13 (0.63-2.02)	2.46 (0.01-566.73)	2.39 (1.23-4.65)	1 (empty)	1 (empty)	1.01 (0.43-2.38)	0.77 (0.30-1.98)	1.76 (0.42-7.36)	1.34 (1.09-1.65)

*Based on patients who had at least one consultation in each period.
